# Closed Genome Sequence of Clostridium botulinum Strain IBCA10-7060 Type Bh

**DOI:** 10.1128/MRA.00383-21

**Published:** 2021-08-26

**Authors:** Nir Dover, Jason R. Barash, John M. Bell, Matthew D. Sylvester, Stephen S. Arnon

**Affiliations:** a Infectious Diseases Laboratory Branch, Division of Communicable Disease Control, Center for Infectious Diseases, California Department of Public Health, Richmond, California, USA; University of Rochester School of Medicine and Dentistry

## Abstract

Clostridium botulinum strain IBCA10-7060 was isolated from a stool specimen from an infant botulism patient and is the only Clostridium botulinum strain known that produces botulinum toxin type H. We present here its 4.09-Mbp closed genome sequence.

## ANNOUNCEMENT

Clostridium botulinum is a Gram-positive anaerobic, spore-forming bacterium with the ability to produce botulinum neurotoxin, the most potent naturally occurring toxin known (1). Botulinum toxin type H was the first new botulinum toxin type to be recognized in more than 40 years (2, 3), and Clostridium botulinum strain IBCA10-7060 is the only *Clostridium* strain known that produces botulinum toxin type H (2–7); this strain also produces botulinum toxin type B (2, 3). Strain IBCA10-7060 was isolated in California from an infant botulism patient, and the stool collection was exempt from institutional review because the testing of specimens from suspected infant botulism patients is required by California law (Health and Safety Code §123704). Previous draft genome sequencing of strain IBCA10-7060 yielded a 4,005,128-bp genome divided into 131 contigs (8).

Strain IBCA10-7060 was subcultured anaerobically from a frozen stock culture on 4% egg yolk agar plates and incubated for 48 h at 35°C. The isolated colonies were inoculated into 20 ml of prereduced Trypticase-peptone-glucose-yeast extract broth, incubated anaerobically for 16 h at 35°C, and harvested by centrifugation at 3,450 × *g*.

DNA was extracted using the Zymo Research (Irvine, CA) Quick-DNA high-molecular-weight (HMW) MagBead kit according to the manufacturer’s instructions. For short-read sequencing (300-bp paired-end reads), libraries were prepared using the KAPA HyperPlus kit (Roche, Basel, Switzerland) and sequenced on a MiSeq instrument (Illumina, San Diego, CA). For long-read sequencing, libraries were prepared using the ligation sequencing kit (SQK-LSK109) and sequenced on the MinION Mk1C device on a FLO-MIN106 flow cell for 24 h (Oxford Nanopore, Oxford, UK).

Guppy version 3.6.0 (Oxford Nanopore) was used for base calling to produce a total of 397,436 long reads with an *N*_50_ value of 31,757 bp. These reads were first filtered using NanoFilt version 2.6 (9) to minimum average read scores of 12, leaving 340,484 reads. The long reads were downsampled four times to produce differing groups of reads with coverage depths of approximately 215-fold. The Illumina short reads were downsampled from 52.8 million reads (26.4 million pairs) to 1.3 million read pairs. All downsampling was done using an in-house python script. Ratatosk version 0.2.3 (10) was run on the filtered long reads to prepolish them and to improve the quality before assembly. Flye version 2.6 (11), Raven version 0.0.7 (12), Redbean (no version available) (13), and Miniasm version 0.3-r179 (14) were each run on all sets of the samples that had been processed using Ratatosk. These data sets were then processed using Trycycler version 0.3.3 (https://zenodo.org/record/4556428#.YFu_R2hKiUl), which clustered, reconciled, and circularized the contigs, thereby producing a single closed consensus genome sequence of 4,090,796 bp with genome coverage of 405-fold. All programs were run with default settings except as noted here: all assemblers were run with 16 threads, Flye and Redbean were given a 4-Mb genome specification, and Flye was given a 1,000 minimum overlap (-m option). Manual inspection indicated a spurious one-base insertion in a homopolymer sequence in *higA*, which was corrected to produce a genome of 4,090,796 bp. The genome sequence was annotated upon submission to NCBI using the Prokaryotic Genome Annotation Pipeline (PGAP) (15).

The closed genome sequence contained 4,090,796 bp (GC content, 28.2%), with 3,714 coding DNA sequences and 9 complete rRNA operons. The genome contained no plasmids. The type H botulinum toxin gene was part of a large 53.7-kb insertion that occurred at a chromosomal location different than that of all other chromosomal or plasmid botulinum toxin gene clusters (3).

### Data availability.

The closed genome sequence of Clostridium botulinum strain IBCA10-7060 has been deposited in GenBank under the accession number CP069280.1, the BioProject accession number PRJNA698972, and the BioSample accession number SAMN17763437. The raw sequences were deposited under the SRA accession numbers SRR14421949 and SRR14421950.
